# Molecular Approaches Fighting Nonsense

**DOI:** 10.3390/ijms222111933

**Published:** 2021-11-03

**Authors:** Ivana Pibiri

**Affiliations:** Dipartimento di Scienze e Tecnologie Biologiche, Chimiche e Farmaceutiche-STEBICEF, Università degli Studi di Palermo, V.le delle Scienze Ed. 17, 90128 Palermo, Italy; ivana.pibiri@unipa.it

Nonsense mutations are the result of single nucleotide substitutions in the DNA that change a sense codon (coding for an amino acid) to a nonsense or premature termination codon (PTC) within the coding region of the mRNA. The severity of nonsense is due to the interruption of protein translation by the production of truncated polypeptides not expressing their function. PTC-containing mRNA can be reduced by a surveillance pathway called nonsense-mediated mRNA decay (NMD). The lack of a functional protein due to a nonsense mutation can result in a number of different genetic disorders, e.g., cystic fibrosis, Duchenne muscular dystrophy, retinitis pigmentosa, congenital blindness, dystonia, spinal muscular atrophy, neurofibromatosis, lysosomal storage disease, Usher’s syndrome, hemophilia, Tay–Sachs disease, Schwackman Diamond syndrome, and several forms of cancer.

Recent research is facing the challenge of targeting the genetic defect itself within the framework of a personalized medicine approach. Most clinical trials have been performed with small molecules that have been developed to target the translation phase and to promote the bypass of PTC, allowing the synthesis of a full-length functional protein, a strategy known as PTC “readthrough” by translational readthrough promoters (TRIDs). In this context, while aminoglicosyde antibiotics are considered outdated for use as readthrough promoters due to their chronic toxicity at higher dosages, Ataluren, a small heterocyclic drug, has been approved as a TRID for Duchenne muscular dystrophy under the trade name Translarna, and ELX02 is a new molecule that is currently in clinical trials for cystic fibrosis caused by nonsense mutations. On the other hand, the correction of the genetic defects at the DNA level has been attempted through modern genome editing strategies such as CRISPR/Cas9.

In this Special Issue, which aims to collect interesting research on this hot topic, both conventional strategies as well as new technologies are discussed [1]. Through the interpretation of experimental and mechanistic findings that were mainly obtained in lysosomal and coagulation disorders, a scenario with potential readthrough-favorable features is proposed to achieve relevant rescue profiles, representing the main issue for the potential translatability of readthrough as a therapeutic strategy [2]. The efficacy of combining readthrough agents and NMD inhibitors has been explored as a potential therapeutic option for treating nonsense mutations [3]. New translational readthrough-inducing drugs (TRIDs) showing high readthrough activity and low toxicity have been explored for the rescue of functional ion channel CFTR, the absence of which is responsible for cystic fibrosis [4]. In the same context, RNA editing approaches such as the CRISPR/dCas13b-based molecular tool “*REPAIRv2*” could be a good alternative to restore the full-length CFTR protein [5]. Inherited retinal diseases (IRDs) are due to nonsense mutations in approximately 12% of all cases. The role of different mutations on ocular channelopathies, the disease mechanism, and the potential pharmacological and therapeutic approaches have been reviewed [6]. In particular, a promising pharmacological approach with TRIDs such ataluren is discussed [7]. Additionally, for primary ciliary dyskinesia (PCD) caused by nonsense mutations, non-aminoglycosides readthrough therapies are an attractive option. A group of chemical compounds with known PTC-readthrough potential (ataluren, azithromycin, tylosin, amlexanox, and the experimental compound TC007) have been investigated, and the tested compounds stimulated PTC-readthrough with lower efficiency than aminoglycosides but had a minimal negative impact on cell viability and function [8]. Importantly, the treatment of inherited bone marrow failure syndromes (IBMFS) due to nonsense mutations in the respective IBMFS-related gene caused by the new generation of nonsense suppressor molecules and their mechanistic roles have been reviewed, with strengths and limitations emerging from preclinical and clinical studies [9].

Although molecular approaches fighting nonsense can be considered as a personalized medicine approach and may, in principle, appear to be a “niche research” topic as they concern “rare” genetic diseases, they can also be exploited for different pathologies. As such, they constitute a general approach to a basic genetic defect, widening the applicative scope and making the exploration of their mechanisms appealing.
